# (Not so) universal literacy screening: a survey of educators reveals variability in implementation

**DOI:** 10.1007/s11881-025-00342-1

**Published:** 2025-10-29

**Authors:** Ola Ozernov-Palchik, Zoe Elizee, Fabio Catania, Meral Hacikamiloglu, Stefanie Shattuck-Hufnagel, Yaacov Petscher, Satrajit Ghosh, John D. E. Gabrieli

**Affiliations:** 1 McGovern Institute for Brain Research, MIT, Cambridge, MA 02139 USA; 2 Wheelock College of Education & Human Development, Boston University, Boston, MA 02215 USA; 3 Research Laboratory of Electronics, MIT, Cambridge, MA 02138 USA; 4 Program in Speech and Hearing Bioscience and Technology, Harvard University, Cambridge, MA 02138 USA; 5 Florida Center for Reading Research, Florida State University, Tallahassee, FL 32306 USA

**Keywords:** Dyslexia, Implementation science, Reading, Survey, Universal screening

## Abstract

Currently, most states in the United States have enacted legislation mandating universal screening for literacy risk in kindergarten through 3rd grade. However, the degree to which these policies translate into consistent, high-quality screening practices remains unclear. In this survey study, we collected responses from a diverse sample of K–3 educators (*N* = 251) across 39 states, representing varied school types, professional roles, and experience levels, to examine the real-world implementation of universal screening. Guided by the Exploration, Preparation, Implementation, and Sustainment (EPIS) framework, we analyzed quantitative and qualitative data to identify real-world factors that could impede the fidelity and effectiveness of screening implementation. We found substantial variability across multiple dimensions of literacy screening implementation. Educators described considerable variation in screener selection, administration practices, testing environments, training quality, scoring accuracy, and the use of results to guide intervention. Notably, many indicated insufficient training and professional development, expressing uncertainty about administering and interpreting screeners, particularly for English language learners. Nearly half also reported the absence of systematic procedures for developing intervention plans, suggesting that many students identified as at risk do not receive appropriate follow-up support. These implementation challenges occurred despite widespread recognition among educators of screening’s importance for early literacy intervention. Educators from lower-socioeconomic status schools reported significantly greater time burdens in conducting screenings and more technology-related challenges compared to their higher-SES counterparts. Without systematic improvements to implementation support and training, current screening initiatives may fail to achieve their intended goal of early identification and intervention for struggling readers.

**Supplementary Information:**

The online version contains supplementary material available at 10.1007/s11881-025-00342-1.

## Introduction

For over two decades, national reading proficiency scores have remained stagnant, with only 30% of students achieving proficiency, a figure that has declined in recent years as racial and socioeconomic disparities continue to widen (National Assessment of Educational Progress, 2024). Despite significant investment, many educational approaches have not produced sustained, large-scale gains. One method strongly supported by research to enhance literacy outcomes is universal literacy screening (Clearinghouse, 2009). Universal screening involves brief assessments of key pre-reading skills associated with future literacy success, aiming to identify students at risk to prevent severe and persistent reading difficulties. Early screening starting in kindergarten is critical because research demonstrates that interventions yield the strongest improvements when implemented in early elementary grades (kindergarten through second grade; Gersten et al., 2020; Neitzel et al., 2022; Slavin et al., 2011; Suggate, 2010; Swanson et al., 1999; Wanzek et al., 2016, 2018).

Consequently, as of 2024, most states mandate universal literacy screening for kindergarten students and require screenings at least twice annually through second grade (National Center for Improving Literacy, n.d.). Data from these screenings are intended to inform instructional responses within a structured approach such as Multi-Tiered System of Supports (MTSS). In MTSS, each successive tier, guided by screening data and ongoing progress monitoring, provides increasingly intensive, individualized interventions. This instructional model is widely implemented to meet diverse student needs (Coyne et al., 2018) and relies fundamentally on accurate identification of at-risk students. Ideally, screening data significantly contribute to enabling most children to reach reading proficiency, maximizing their academic potential.

The policy push toward universal screening has sparked national debates centered on the theoretical foundations of instructional materials (e.g., whole language vs. phonics) and the psychometric properties of screening assessments, particularly regarding their accuracy in classifying at-risk students (Glover & Albers, 2007; Johnson et al., 2009; Petscher et al., 2011). Amid these discussions, however, a crucial factor often receives less attention: variability in screening implementation practices. Although screening design and validation receive emphasis, practical decisions regarding administration, scoring, and follow-up support are equally influential in determining whether students receive necessary assistance (Komesidou et al., 2022).

Federal policies typically outline broad requirements for evidence-based screening and intervention but often leave implementation details unspecified. Similarly, many states issue guidelines for selecting appropriate screening and instructional tools to bridge federal mandates and classroom realities (Gearin et al., 2022), yet these guidelines grant significant latitude to districts and schools in practical implementation. Research consistently shows that these implementation decisions significantly influence educational outcomes (e.g., Al Otaiba & Fuchs, 2006; Durlak & DuPre, 2008; Kaderavek & Justice, 2010; Vaughn et al., 2015). Educational programs rarely operate exactly as intended, and deviations often compromise effectiveness. Universal screening is similarly vulnerable to implementation challenges, with variability occurring at multiple stages, including teacher training, administration practices, resource allocation, and follow-up processes. Each deviation can undermine screening effectiveness in identifying and supporting at-risk students. In this paper, we examine educator-reported factors influencing universal screening implementation and effectiveness in real-world settings.

### Screener implementation

Effective universal screening implementation requires careful attention to multiple interconnected elements, which can be systematically examined through the Exploration, Preparation, Implementation, and Sustainment (EPIS) framework (Moullin et al., 2019; Sanetti & Luh, 2019). Originally developed for healthcare settings and subsequently adapted for educational contexts (Aarons et al., 2011; Sanetti & Luh, 2019), the EPIS framework provides a structured approach to examining the acceptability, execution, and sustainment of educational practices within complex systems, addressing factors at both organizational and individual levels. While EPIS traditionally emphasizes temporal sequencing, we employ it as a multi-dimensional framework where each dimension represents essential implementation considerations. Exploration assesses organizational readiness and identifies potential barriers such as resource limitations or competing priorities. Preparation establishes a robust foundation through comprehensive educator training and capacity building. Implementation focuses on adherence to screening procedures and protocol fidelity, while Sustainment ensures long-term quality maintenance and systematic use of screening data to inform ongoing interventions.

A critical consequence of variability across these EPIS components is the extent to which publisher-recommended administration and scoring procedures, commonly referred to as fidelity of implementation, are adhered to. We can consider high classification accuracy in school-based screening as a target. In validation studies, this level of accuracy is achieved under ideal conditions, including standardized procedures, trained administrators, and optimal technical quality. Achieving comparable results in real-world settings requires effective execution across all EPIS levels. However, each deviation, whether due to inadequate training, environmental disruptions, or erosion of practices, shifts actual accuracy away from this ideal, undermining correct identification of at-risk students. This variability is particularly problematic given that baseline accuracy rates for most screeners already fall short of perfection (Petscher, 2024). For students with disabilities, compounded deviations across the EPIS phases can mean the difference between timely support and being overlooked entirely. Although substantial research has focused on the fidelity of reading intervention delivery (Reed et al., 2014; Sanetti et al., 2019), comprehensive attention to the implementation of screener assessments from initial adoption through sustained practice remains limited.

Inconsistencies in educator preparation, administration procedures, and long-term quality assurance can lead to student misidentification and ineffective instructional decision-making (Reed & Sturges, 2013). Such ineffective decision-making manifests in teachers’ reliance on intuitive rather than data-driven instructional modifications, failure to design meaningful instructional changes, and delayed intervention provision where at-risk students experience substantial waiting periods before receiving targeted support (Al Otaiba & Fuchs, 2002; Fuchs & Fuchs, 2006; Fuchs et al., 2003; Vaughn et al., 2015). Persistent equity concerns include both over-identification, where students are incorrectly flagged as at-risk, and under-identification, where students who will ultimately struggle are overlooked during initial screening. These identification disparities disproportionately affect students from certain demographic groups and may arise at any phase of the EPIS framework, from initial resource allocation to ongoing monitoring and support (Cassidy et al., 2023; Papandrea et al., 2023; Schelbe et al., 2022; Skrtic et al., 2021). Without addressing these practical implementation factors, universal screening systems risk falling short of their promise, failing to inform robust instructional decision-making and leaving too many children without the support they need.

Existing research on universal screening has largely focused on statistical metrics of screeners (e.g., Edwards et al., 2022), the intersection between screening-based policies and implications for screening-based practices (e.g., Gearin et al., 2022), or practical considerations regarding screening and early identification (e.g., Gaab & Petscher, 2022). An overlooked segment of research in this area includes educators’ perspectives, despite their central role in implementation. Several studies have explored educators’ attitudes toward specific screening tools and highlighted barriers or facilitators to effective implementation (Hoffman et al., 2009; Komesidou et al., 2022; Rowe et al., 2014). Komesidou et al. (2022) specifically identified educator-reported factors across five domains: screener features, preparation, administration, user demands, and interpretation of results. This study underscored the importance of structured implementation strategies, robust training, and researcher-school collaboration to ensure effective screening and intervention. The current study extends and replicates this work, employing a mixed-methods approach to evaluate these factors in a random sample of practitioners directly involved in screening from kindergarten through third grade classrooms.

Using the EPIS framework as a structured lens through which to analyze these multi-level implementation factors systematically, this study addresses four research questions: (1) Exploration—What organizational and contextual factors characterize current universal screening practices? (2) Preparation—To what extent do educators receive adequate training and feel prepared for screener administration? (3) Implementation—What barriers do educators encounter during administration, and how do these vary by school context? (4) Sustainment—How effectively do screening practices translate into meaningful interventions and equitable outcomes for all students? By examining these implementation factors through educator perspectives, this study aims to identify sources of variability that may compromise screening effectiveness and inform strategies for improving fidelity and equity.

## Methods

### Participants and recruitment

Participants (*N* = 251) were kindergarten through third-grade educators directly involved in the administration of universal literacy screening across 39 states (see SI). They were recruited through social media platforms (Twitter, LinkedIn, Facebook) via the researcher’s professional network and literacy organizations such as the National Center for Improving Literacy and Florida Center for Reading Research that posted the survey to their networks. Additionally, we compiled a list of school districts in rural and urban U.S. communities and sent emails to administrators whose contact information was publicly available.

### Instrument

The 61-item survey was developed by the author team with iterative input from several speech-language pathologists (SLP) and literacy specialists to ensure its relevance and clarity. Survey development was systematically guided by the EPIS framework (Sanetti & Luh, 2019), with constructs defined as follows: Exploration (organizational readiness and contextual factors), Preparation (training quality and educator confidence), Implementation (real-world barriers and fidelity challenges), and Sustainment (long-term viability and intervention effectiveness). Response scales were selected based on established survey design principles (Fowler, 2014), using Likert scales for confidence and quality measures, frequency scales for implementation challenges, and categorical options for demographic and contextual variables. To establish a baseline for key implementation knowledge, three highly experienced SLP literacy specialists from the authors’ professional network completed the survey. Their responses provided a foundational reference for understanding standard administration procedures and expert perspectives on key challenges in literacy screening rather than serving as a validation process. Several survey questions were adapted from Komesidou et al. (2022), which examined barriers and facilitators in implementing screeners for developmental language disorder and dyslexia in school settings and from Al Otaiba et al. (2019), which examined teacher knowledge about MTSS (formerly “response to intervention”). Item mapping to EPIS constructs is reported in the Results section and aligns with the framework’s construct definitions.

### Procedures

The anonymous survey was designed and administered via Qualtrics, a web-based platform. Participants received an electronic survey link and/or QR code for access. Before beginning, they completed a consent form outlining the study’s purpose, confidentiality measures, and potential risks and benefits. No incentives or reimbursements were provided for participation. The survey took approximately 10–20 min to complete, and responses were collected over a 6-month period.

### Analysis

#### Missing data

We conducted comprehensive missing data analysis on the survey dataset (49 variables) including the following: (1) descriptive statistics by variable and question position, (2) chi-square independence tests across variable pairs, (3) Spearman correlation analysis between question order and missing rates, and (4) demographic sensitivity analysis by socioeconomic status. Based on findings, we implemented multiple imputation using MICE with 20 imputations, excluding variables with > 80% missing data, and incorporating survey fatigue indicators as auxiliary variables. However, imputation was unsuccessful for several items with multiple response categories (five to eight levels) where the combination of moderate-to-high missingness (27–50%) and numerous response options created sparse data conditions that prevented reliable imputation.

#### Descriptive summary

To provide a comprehensive overview of literacy screening implementation conditions, we generated frequency tables for key variables categorized under the EPIS framework. This approach allowed us to systematically quantify trends in educator preparedness, implementation challenges, and sustainability factors.

For Exploration, we examined the overall distribution of our sample, contextual readiness, including the variations in district policies. Preparation focused on respondent perceptions of training quality, perceived sufficiency of training time, and the extent to which educators felt confident administering screeners. Implementation encompassed real-world barriers reported by respondents, such as environmental disruptions and technical difficulties. Lastly, Sustainment assessed the long-term viability of literacy screening, including confidence in data interpretation, reported adherence to standardized screening procedures, alignment with MTSS, and perceived effectiveness in identifying at-risk students.

Frequencies and proportions were calculated for each category, summarizing response distributions across key dimensions of literacy screening. The proportion of “no response” entries is included in supplemental information (SI) materials to ensure transparency in reporting and to highlight areas where educators may have lacked information or chose not to respond. These descriptive statistics establish a foundational understanding of implementation variability.

#### Socioeconomic effects on implementation

We examined the relationship between socioeconomic status (SES) and the implementation conditions of literacy screening. SES was determined by the percentage of students receiving free or reduced-price lunch and categorized into high SES (0–10% or 10–30%) and low SES (30–60% or 60 + %). Implementation conditions were assessed across multiple dimensions, including environmental factors, technical difficulties, and interruptions. Responses were categorized as positive or negative based on their implications for screening effectiveness. Responses indicating minimal disruptions, positive conditions, or no challenges (e.g., “Quiet,” “Very quiet,” “Beneficial,” “No issues”) were categorized as positive, while responses indicating difficulties or disruptions (e.g., “Noisy,” “Very noisy,” “Challenging,” “Technical difficulties”) were categorized as negative. To assess differences in implementation conditions by SES, Pearson’s chi-square tests were conducted. Only responses that provided valid categorical data (excluding “Unknown” or “No response”) were included in the analysis.

#### Open-ended responses

Because automated thematic analysis using large language models (LLMs) has been shown to yield results comparable to human analysis (Bareh, 2025) while requiring fewer resources, we opted to use GPT-4.5 to perform inductive thematic analysis (ITA) on open-ended survey responses. Our objective was to systematically identify recurring themes related to literacy screening practices, educator preparedness, implementation challenges, and sustainability. We followed Braun and Clarke’s (2006) six-phase framework, prompting GPT-4.5 to execute each step in sequence: (1) familiarization with the data, (2) generation of initial codes, (3) theme identification, (4) theme review, (5) theme definition and naming, and (6) report production. GPT-4.5 was selected for its state-of-the-art performance in language understanding and reasoning at the time of the study.

To promote consistency and mitigate contextual bias, the model was instructed to analyze each response independently, without access to prior coding decisions. This approach enforced a uniform coding protocol across all samples. To ensure accuracy and face validity, the final themes and coding structure generated by the model were reviewed by a human expert in qualitative methods. By adopting a fully automated, AI-led workflow with human verification, we aimed to reduce subjective bias, enhance reproducibility, and minimize the annotation burden typically associated with qualitative coding.

We additionally assessed qualitative response representativeness by comparing demographic composition of respondents providing open-ended responses to the overall sample. Proportionality ratios near 1.0 indicate representative patterns; ratios substantially above/below 1.0 suggest demographic bias. Chi-square tests evaluated statistical significance. Of 307 respondents, 183 (59.6%) provided at least one qualitative response (with an average 11% of responses per question). Proportionality analysis across five demographic dimensions revealed representative patterns for SES (ratios 0.83–1.11, *p* = 0.083) and ELL population (0.98–1.06, *p* = 0.67), professional role (0.61–1.36, *p* = 0.098), school type (0.85–1.38, *p* = 0.652), and district type (0.95–1.40, *p* = 0.546), confirming absence of systematic bias in qualitative responses.

## Results

### Missing data

Missing data affected 29.74% of all possible data points (3614 out of 12,152) across 41 of 49 variables (not including open-ended questions), with individual missing rates ranging from 3.2 to 83.47% and 170 unique missing patterns indicating systematic rather than random non-response. Comprehensive missing data procedures and results are reported in the SI missing data section. Moderate correlation between question order and missing rates (ρ = 0.58, *p* < 0.001) demonstrated survey fatigue as the primary missing data mechanism, with early questions (1–10) averaging 26.1% missing versus 40.7% for late questions (31–49), indicating systematic abandonment due to respondent burden (SI Table 1). Chi-square tests across 819 variable pairs showed 86% had dependent missingness (*p* < 0.05), providing strong evidence against Missing Completely At Random (MCAR), with dependencies reflecting position-based abandonment rather than content-specific patterns. Missing patterns showed no significant relationship to demographics (*p* > 0.16) and survey fatigue effects were consistent across Free/Reduced Lunch categories (*p* = 0.290), with similar fatigue effects across all groups (25.1–40.1 percentage point drops), indicating survey position rather than respondent characteristics drives systematic missingness (SI Table 1). Based on Missing At Random (MAR) classification, we implemented multiple imputation using MICE with 20 imputations.

### Exploration

This section characterizes the landscape of universal literacy screening implementation in the current sample as a small window in the at-large universal literacy screening landscape in the United States, examining organizational contexts, implementer characteristics, and student populations served. This section provides essential context for understanding the implementation experiences, resource needs, and barriers described in the other sections.

Survey respondents (Table 1) were all directly involved in administering universal screeners to students in kindergarten through third grade and represented a wide range of professional experience. The majority of respondents were literacy specialists (36%), classroom teachers (25%), or special education teachers (16%) (Table 1). Respondents who selected “Other” (15%) described diverse roles in literacy education, including dyslexia specialists, interventionists, administrators, and higher education professionals, highlighting the broad expertise supporting universal screening.

**Table 1:** Exploration: educator roles, school contexts, and screening variability

Response	Count	Percent
**What is your role?**		
Classroom Teacher	62	25.00%
Special Education Teacher	39	15.73%
Literacy Specialist/Coach	90	36.29%
Speech Language Pathologist	5	2.02%
School Counselor/Psychologist	3	1.21%
School Administrator	9	3.63%
Adult Volunteer	4	1.61%
Other	36	14.52%
**What type of school do you work in?**		
Public	210	84.68%
Charter	9	3.63%
Private	17	6.85%
Diocese/Parochial	2	0.81%
Other	10	4.03%
**What type of district do you work in?**		
Urban	55	22.18%
Suburban	117	47.18%
Rural	60	24.19%
Other	16	6.45%
**What grade(s) do you teach?**		
Kindergarten	16	6.45%
Kindergarten, 1 st grade, 2nd grade	14	5.65%
Kindergarten, 1 st grade, 2nd grade, 3rd grade	25	10.08%
1 st grade	36	14.52%
1 st grade, 2nd grade	17	6.85%
1 st grade, 2nd grade, 3rd grade	33	13.31%
2nd grade	25	10.08%
2nd grade, 3rd grade	24	9.68%
3rd grade	58	23.39%
**How many years of experience do you have?**		
0–1 years	5	2.02%
1	11	4.44%
2	47	18.95%
2–5 years	21	8.47%
3	23	9.27%
4	68	27.42%
6–10 years	25	10.08%
11 + years	48	19.35%
**What percentage of the students in your school are bi/multilingual?**		
0–10%	100	40.32%
10–30%	65	26.21%
30–60%	28	11.29%
60 + %	24	9.68%
Unknown	31	12.50%
**What is the percentage of free or reduced lunch students in your school?**		
0–10%	47	18.95%
10–30%	38	15.32%
30–60%	50	20.16%
60 + %	78	31.45%
Unknown	35	14.11%
**What percentage of the students in your school are ELL?**		
0–10%	117	47.18%
10–30%	63	25.40%
30–60%	36	14.52%
60 + %	16	6.45%
Unknown	16	6.45%
**How often is the literacy screener administered throughout the school year?**		
Once at the beginning	13	5.24%
Twice (beginning and end)	13	5.24%
Three times (beginning, middle, and end)	200	80.65%
Other	22	8.87%

Most respondents worked in public school districts (85%) across urban, suburban, and rural settings, although private (7%) and charter (4%) schools were also represented. The districts were socioeconomically and linguistically diverse, with 10–60% of students qualifying for free or reduced lunch in 67% of schools, and approximately 46% of schools serving at least 10% English language learners (ELL). Thus, the survey reflects a wide and representative range of professional perspectives.

Practitioners reported 60 unique screener combinations from 249 survey responses. DIBELS 8th Edition was the most frequently used single tool (29.3%), followed by mCLASS from Amplify (18.9%) and i-READY from Curriculum Associates (18.5%) (SI Table 4). Common combinations included DIBELS 8th Edition and i-READY, DIBELS 8th Edition and STAR, and mCLASS and STAR (SI Table 5). Thirty-six respondents cited additional screeners, including Aimsweb, Istation Reading ISIP, and Clay’s Observation Survey. Assessment formats were predominantly digital (75%), with traditional paper–pencil methods comprising 15% of responses. Most practitioners (81%) reported testing three times per year.

### Preparation

This section examines the training experiences that prepared educators to implement universal literacy screening and educators’ perceptions of the training they received and their preparedness. Understanding how educators develop the knowledge, skills, and confidence needed for effective screening administration is critical for successful implementation.

Responses (Table 2) showed wide variability in training duration. Although the publishers of the most frequently administered assessment, DIBELS, recommend 4–8 hours of training (National Center on Intensive Intervention, n.d.), 75% of educators administering this assessment reported receiving less than 3 hours of training with 44% reporting less than 1 hour or no training at all.

**Table 2:** Preparation: educator training experiences and confidence in screening

Response	Count	Percent
**How confident did you feel to administer the literacy screener?**		
Not at all confident	20	8.06%
Slightly confident	26	10.48%
Somewhat confident	71	28.63%
Quite confident	88	35.48%
Extremely confident	43	17.34%
**How much training did you receive on screener administration?**		
Less than 1 h	61	24.60%
1–3 h	78	31.45%
3–8 h	28	11.29%
More than 8 h	6	2.42%
No training	47	18.95%
Ongoing or continuous training	28	11.29%
**Describe the time you were given to complete training**		
Insufficient	71	28.63%
More than sufficient	31	12.50%
Sufficient	146	58.87%
**What mode of training was provided?**		
In-person only	70	38.89%
Hybrid (in-person + virtual) only	29	16.11%
Virtual only	22	12.22%
Asynchronous only	17	9.44%
In-person + asynchronous	9	5.00%
In-person + virtual	6	3.33%
In-person + hybrid	3	1.67%
Other mixed modes	24	13.33%
*No response (out of total sample)*	68	27.42%
**How would you rate the quality of the training provided?**		
Very poor	2	0.81%
Poor	38	15.32%
Average	84	33.87%
Good	84	33.87%
Excellent	19	7.66%
Other	21	8.47%
**Were you given an opportunity to practice administering the screener?**		
I don’t remember	24	9.68%
No	114	45.97%
Yes	110	44.35%

Almost half (46%) of respondents said they had no opportunity to practice before screening their first child. Although many (68%) rated their training as “average” (34%) or “good” (34%), 16% felt it was “poor” or “very poor.” Only 52% of the respondents reported feeling confident by the end of their initial training, while nearly 47% felt only “somewhat confident” (29%), “slightly confident” (10%), or “not at all confident” (8%).

### Training quality

Qualitative analysis of open-ended responses provided further insights into the strengths and challenges of educator training experiences (SI Table 6, SI Table 7). Educators highlighted the benefits of structured professional development, refresher sessions, and self-paced online resources and hands-on practice for building competence and confidence. However, many reported insufficient preparation, particularly noting “no onboarding for new hires” and describing initial training as “a quick overview” with “all meaningful learning on my own.” Participants consistently emphasized the need for ongoing training during school hours, with one stating “the district needs to provide us with ongoing training during school hours!” District-level training often prioritized upper-elementary benchmarks, leaving K–3 teachers to self-teach or seek informal peer support when facing difficulties. Some educators felt specifically unprepared to interpret results from phonological awareness measures or nonsense word tests, often employed as well validated measures of pre-reading language skills that are predictive of future reading outcomes. Training quality concerns were evident in responses noting trainers who were “poor for interpretation of results” and had “never used the screener/program.”

### Professional development recommendations

Educators emphasized the critical need for structured, continuous professional development and improved onboarding processes to enhance literacy screening practices. They recommended regular refresher training sessions, interactive workshops, video tutorials, and peer-led coaching sessions, with content closely aligned with instructional goals. A prevalent theme was insufficient preparation, particularly regarding interpreting phonological awareness measures and nonsense word tests, with respondents frequently citing inadequate district-level support (SI Table 6). Many educators expressed frustration with training that focused primarily on technical setup rather than meaningful data interpretation, noting that they “were trained how to set up screeners and where to retrieve the data, but not on what exactly the data shows, what each category means, or even what is specifically asked of students on the test” (SI Table 7).

### Practice opportunities and feedback systems

Experiences with practice and feedback varied significantly (SI Table 8). Some educators received valuable guided practice with “experienced tester sat with me and gave feedback” and targeted feedback from experienced observers or peers. Yet, many educators lacked formal feedback, with responses simply stating “no feedback” and relying instead on self-guided practice or online demonstration tools with minimal evaluation. Additionally, specialized training in early literacy screening was frequently superficial, increasing educators’ reliance on informal peer support.

The importance of hands-on practice with structured feedback emerged as a central theme, with educators emphasizing that training should be embedded within regular school hours to ensure consistency and effectiveness. Successful models included collaborative approaches where literacy coaches provided “colleagues with the opportunity to practice administering “the screener in an ‘I do, we do, you do.’” format and group practice sessions where teams “met in the library and did a few practice screeners together, graded them, and questions were answered.” Peer observation and feedback systems also proved valuable, with educators describing experiences where they “watched a coworker administer it, then administered it while she watched and she gave feedback afterwards.”

### Administrator confidence levels

Administrator confidence levels varied with their backgrounds and preparation experiences (SI Table 9). Those with strong educational foundations expressed high confidence, with responses such as “I have a masters in reading” and “I’ve been an interventionist for 30 years.” However, many described initial uncertainty and anxiety, with educators stating “I was worried I wasn’t doing it correctly at first” and “it always takes some practice getting into the routine.” Specific administrative and scoring challenges were frequently mentioned, particularly noting “NWF felt hard to administer correctly” and concerns about “MCLASS particular ways you must mark mistakes.”

### Implementation

This section examines the practical aspects of administering literacy screeners, including logistical challenges, environmental factors, technical difficulties, and time demands.

Educators reported (Table 3) substantial variability in screening conditions, which according to them influenced both student engagement and the accuracy of results. These implementation challenges encompassed environmental factors, technical difficulties, timing constraints, and administrative barriers.

**Table 3:** Implementation: Variability in screening conditions and fidelity challenges

Response	Count	Percentage
**Was the screening administered in a familiar environment for students?**		
No, different location	92	37.10%
Yes, in home	8	3.23%
Yes, in regular classroom	148	59.68%
**How would you rate the level of noise in the environment?**		
Very quiet	30	12.10%
Quiet	115	46.37%
Moderate	83	33.47%
Noisy	19	7.66%
Very noisy	1	0.40%
**Do you ever experience unexpected interruptions during the screener?**		
No	46	18.55%
Yes	202	81.45%
**At least 1 interruption**		
0–10%	61	38.13%
10–30%	68	42.50%
30–60%	18	11.25%
60 + %	13	8.13%
*No response (out of total sample)*	89	35.74%
**Do you ever experience technical difficulties during the screener?**		
No	115	46.37%
Yes	133	53.63%
**At least 1 technical issue**		
0–10%	63	58.88%
10–30%	34	31.78%
30–60%	8	7.48%
60 + %	2	1.87%
*No response (out of total sample)*	142	57.03%
**Do you ever experience issues with clarity/audibility of multimedia components?**		
No	167	67.34%
Yes	81	32.66%
**At least 1 clarity/audibility issue**		
0–10%	27	64.29%
10–30%	7	16.67%
30–60%	8	19.05%
*No response (out of total sample)*	207	83.13%
**How does the mode of administration impact the screening process?**		
Beneficial	65	26.21%
No challenge	84	33.87%
Minor challenge	45	18.15%
Moderate challenge	42	16.94%
Significant challenge	12	4.84%
**Approximately how long does screener administration take?**		
Less than 15 min	85	34.27%
15–30 min	82	33.06%
30–60 min	51	20.56%
60 + min	30	12.10%
**How does the duration of the screening process impact its implementation?**		
Beneficial	6	2.42%
No challenge	58	23.39%
Minor challenge	84	33.87%
Moderate challenge	69	27.82%
Significant challenge	31	12.50%
**How does family involvement during the screening impact implementation?**		
Beneficial	1	2.44%
No challenge	12	29.27%
Minor challenge	10	24.39%
Moderate challenge	9	21.95%
Significant challenge	9	21.95%
*No response (out of total sample)*	208	83.53%
**Do you ever experience difficulties in scoring?**		
Did not score myself	52	20.97%
No difficulties encountered	93	37.50%
Minor difficulties, but manageable	80	32.26%
Significant difficulties	8	3.23%
Other	15	6.05%
**What challenges have you encountered?**		
Difficulty in interpreting screener results	5	6.41%
Insufficient training or support	3	3.85%
Lack of resources for follow-up interventions	6	7.69%
Lack of time during the school day	16	20.51%
Technical issues with the screener tool	9	11.54%
Difficulty in interpreting screener results + lack of resources for follow-up interventions	2	2.56%
Lack of resources for follow-up interventions + resistance from parents	2	2.56%
Lack of time + insufficient training or support	5	6.41%
Lack of time + lack of resources for follow-up interventions	8	10.26%
Lack of time + lack of resources for follow-up interventions + resistance from parents	5	6.41%
No challenges	17	21.79%
*No response (out of total sample)*	68	46.58%
**How was the literacy screener administered?**		
Hybrid	8	3.23%
In-person	228	91.94%
Remote/virtual	6	2.42%
Other	6	2.42%

### Environmental factors and testing conditions

One key factor affecting implementation was student familiarity with the testing environment. The majority (60%) administered the screener in a regular classroom, while 37% used alternative settings such as hallways, libraries, specialized intervention rooms, small group settings, or computer labs. About 58% of responders indicated that the environment for screening was “very quiet” (12%) or “quiet” (46%). However, the majority of educators (81%) reported experiencing interruptions during screening sessions. Among those experiencing disruptions, 62% reported disruptions in more than 10% of their sessions.

In open-ended responses, environmental considerations emerged as critical factors affecting both student comfort and the perceived assessment validity (SI Table 10). Many participants emphasized the importance of controlled environments for accurate results, with one noting that “conducting this assessment in person provides accurate results.” Many participants reported successful assessments in familiar environments, noting “students were happy to come and it was actually less distracting.” However, shared or busy spaces created significant challenges, with educators describing “distractions in the hallway…other kids pass by” and “resource room shared and can be loud.” Educators noted that students were easily distracted in less controlled environments, particularly in hallways where high noise levels and unfamiliar surroundings made it difficult to focus, with responses noting “new space, new distractions”; “apprehension due to the novel environment” and “students interested in surroundings they hadn’t seen before.” Some administrators developed specific comfort-enhancing strategies, with one stating “I use calming colors and stuffies in my office” and another noting the importance to “orient the child to the environment…answer questions before we begin.”

### Screening duration and student engagement

Screening duration varied considerably, with 34% of respondents reporting that screenings took less than 15 min, 33% indicated durations between 15 and 30 min, and 12% reported screenings lasting over an hour. The majority felt that screening duration had little impact on scheduling, while just over a third noted that longer assessments negatively affected student engagement and instructional planning. Only 23% reported no challenges related to screening duration, while 34% reported minor challenges, and the remainder indicated moderate or significant difficulties.

Open-ended responses highlighted that assessment duration significantly impacted implementation success, particularly regarding student engagement (SI Table 11). Participants consistently noted that “engagement is negatively impacted when the assessment is too long” and that “many children need multiple sessions due to lack of attention.” Educators conducting lengthier screenings frequently observed student fatigue and disengagement, with responses noting students “get very bored which negatively impacts how well they do” and “children get bored taking iReady so they will often just click through.” Scheduling challenges compounded these issues, with educators describing “two-week window is stressful…scramble to do screenings” and noting “it can be challenging to find screening time with a busy caseload.” Individual student differences required flexible approaches, with responses indicating “students with language disorders, autism, ADHD sometimes take longer” and “some kids sustain attention better than others,” necessitating accommodations that could further complicate scheduling.

### Technical difficulties and hardware challenges

Technical difficulties during screenings were common, with 54% of educators reporting at least one issue. Among these, 41% experienced technical difficulties in more than 10% of sessions. Issues with clarity and audibility of multimedia components were also reported by 33% of respondents. Of these clarity issues, 36% occurred in more than 10% of sessions.

Technology needs reflected both infrastructure limitations and desires for enhanced functionality (SI Table 12). Many participants requested basic device improvements, specifically seeking “iPads available to all teachers” and “updated Chromebook.” The most frequently cited problems included unreliable internet connections, software glitches, and device-related obstacles, with educators noting “networks are not able to handle multiple classes giving screeners at the same time” and “connectivity can be an issue.” Audio quality emerged as a persistent concern, with requests for “high-quality headphones” and “microphones or reliable audio pickup.” Some participants envisioned enhanced digital assessment capabilities, hoping for systems where it “would be nice if computer scored oral responses” and “a true application that learns from student answers” with “more detailed analysis of student errors.” Interestingly, some educators expressed preference for traditional methods, wanting to “return to paper/pencil” and noting “less technology is wanted for K-2!”.

### Scoring challenges and accuracy concerns

Regarding scoring, 38% reported no difficulties, 32% reported minor manageable difficulties, and 3% faced significant difficulties. Approximately 21% did not score assessments themselves. Primary challenges included insufficient time during the school day (21%), technical issues (12%), and lack of resources for follow-up interventions (8%).

While automated scoring provided relief for many administrators, with open-ended responses noting “the assessment is automatically scored through the system,” several scoring challenges persisted (SI Table 13). Real-time scoring proved particularly difficult, with educators stating “if a student reads quickly…it can be hard to keep up” and noting “we mostly audio record to ensure validity.” Furthermore, responses highlighted that while automated scoring streamlined the process for many educators, concerns remained about accuracy and the inability to account for nuanced student errors, with educators noting “there is no way to correct answers for students with language differences” and “it is extremely difficult to mark errors in real time on the non-word fluency portion.” Task-specific difficulties were common, with participants specifically mentioning “Phoneme Segmentation task and the nonsense word fluency task are difficult to score.” Human variability in scoring created consistency concerns, with responses noting “some teachers are more lenient than others” and expressing uncertainty about “judgment calls regarding cut scores.”

### Remote and hybrid administration challenges

The majority of respondents (92%) administered literacy screeners in person, while a small proportion (6%) conducted screenings remotely or in a hybrid format. While in-person screening was generally preferred, some educators who conducted assessments remotely or in hybrid settings noted additional challenges, such as technical difficulties, increased distractions at home, and difficulty monitoring student engagement.

Limited experience with remote or hybrid administration was common (SI Table 14), with many responses indicating “no hybrid or virtual option” in their contexts. Where family involvement occurred, it often created validity concerns through parental interference, with educators noting “parents help students by giving answers” and “sometimes parents interfere by giving prompts or cues.” Several respondents raised concerns about family involvement, explaining that parents sometimes interfered with the screening process by assisting their children or creating additional distractions, with responses noting “parents intervening and making results invalid.” Environmental challenges at home included “constant distraction—TV, siblings, parents interrupting,” while parent understanding of assessment protocols was often insufficient, with one response noting “parents didn’t understand the importance of the lesson and testing,” leading to compromised results and “scores were extremely inflated.”

### Time management and resource allocation

Many educators struggled to fit assessments into their instructional schedules, often having to split screenings into multiple sessions, causing disruptions, with educators noting “getting sub coverage to complete assessments” and “frustration from classroom teachers that literacy specialists aren’t providing services because they’re testing” (SI Table 11). Schools providing additional staffing support experienced fewer issues compared to those relying solely on classroom teachers.

Despite time demands, many participants viewed screening as worthwhile, with responses stating “the time is worth it. The info is valuable” and “the time used on assessments is valuable to instruction” (SI Table 15). While educators acknowledged the value of literacy screeners, many found the administration process time-consuming, often disrupting instruction and interventions. Administrative and logistical responsibilities were substantial, with educators describing being “responsible…to prepare materials, make schedules, assess, score, interpret results.” Some schools addressed time concerns through delegation, with responses noting “someone else administers and scores” or “it’s all computerized,” while others noted efficiency improvements, stating “our current screener takes less than half the time our previous screener took” and “DIBELS used to take time, now through mClass there’s no scoring time.” For some educators screening tasks extended into personal time, with one noting “I do a lot of this at home so it takes away from my family.”

#### SES factors in implementation

**Fig. 1:**
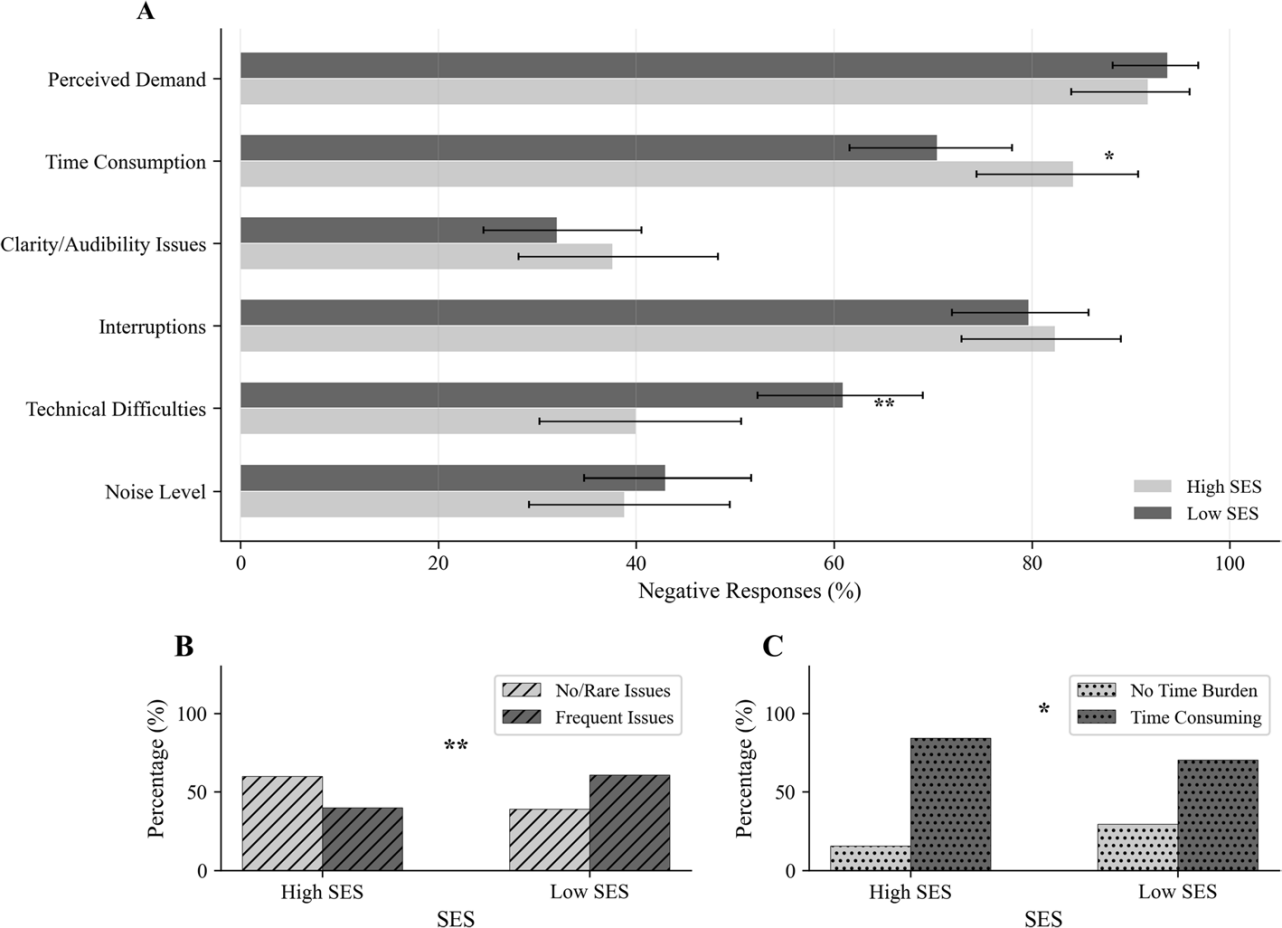
Differences in implementation conditions by school SES. **A** Negative responses by condition (higher bars = more negative), with Wilson 95% confidence intervals; **B** technical difficulties (no/rare issues vs frequent issues); **C** time consumption (no time burden vs time consuming). Variables include Noise Level, Technical Difficulties, Interruptions, Clarity/Audibility Issues, Duration Impact, and Time consumption (task-specific time burden). Coding mirrors the original: for Time Consumption, “No, it did not take time away …” = Positive; any ”Yes, … was most time-consuming” = Negative; “Other” is Neutral (excluded). *p < .05, **p < .01

The analysis revealed that technical difficulties during screening varied significantly by SES (χ^2^(1) = 8.16, *p *= 0.004; see Fig. 1), indicating that educators in lower-SES settings were more likely to report such challenges. In contrast, perceived time consumption of the screening process was lower in lower-SES settings (χ^2^(1) = 4.02, *p *= 0.045), suggesting that these schools were more likely to report that the screening did not take time away from other responsibilities. No other implementation conditions showed statistically significant differences by SES (all *p* ≥ 0.40).

### Sustainment

Sustainment refers to the long-term viability and effectiveness of literacy screening, ensuring that identified students receive appropriate interventions, that screening practices remain equitable and consistent over time, and that educators are adequately supported in implementing and interpreting results (Table 4).

**Table 4:** Sustainment: gaps in data use, intervention access, and equity

Response	Count	Percentage
**Do you feel all your students have equal opportunity to qualify for intervention?**		
No	108	43.55%
Not sure	22	8.87%
Yes	118	47.58%
**Do you feel there is a systematic, formal problem-solving process for an intervention plan?**		
No	117	47.18%
Not sure	22	8.87%
Yes	109	43.95%
**How confident do you feel interpreting data from the literacy screener?**		
Not at all confident	2	0.81%
Slightly confident	18	7.26%
Somewhat confident	39	15.73%
Quite confident	110	44.35%
Extremely confident	79	31.85%
**Across a school year, what percentage of qualified screening results lead to proper intervention?**		
10–30%	69	27.82%
30–60%	53	21.37%
60 + %	86	34.68%
Not sure	40	16.13%
**Does the screener take valuable time away from your other responsibilities?**		
No, it did not take time away	57	22.98%
Preparing materials was most time-consuming	45	18.15%
Administering assessment was most time-consuming	50	20.16%
Scoring was most time-consuming	33	13.31%
Multiple aspects equally time-consuming	38	15.32%
Other	25	10.08%
**How often do you retest due to doubts about literacy screening accuracy?**		
Never	15	6.05%
Rarely	111	44.76%
Sometimes	94	37.90%
Often	27	10.89%
Always	1	0.40%
**How often do you adjust literacy screenings based on student needs?**		
Never	30	12.10%
Rarely	59	23.79%
Sometimes	79	31.85%
Often	47	18.95%
Always	33	13.31%
**How confident are you using screening data for tier 1–tier 3 instruction?**		
Not sure	72	29.03%
Not at all confident	27	10.89%
Slightly confident	19	7.66%
Somewhat confident	36	14.52%
Quite confident	58	23.39%
Extremely confident	36	14.52%
**Do you feel future directions and decisions can be made in a timely manner?**		
No	75	30.24%
Yes	173	69.76%
**Overall, how would you describe the demand of the entire process?**		
Not at all demanding	17	6.85%
A little demanding	64	25.81%
Somewhat demanding	139	56.05%
Extremely demanding	28	11.29%
**Are ELL students administered the universal screener?**		
No	19	7.66%
Yes	229	92.34%
**In my view, ELLs cannot be reliably screened for risk of reading difficulties**		
True	57	22.98%
False	191	77.02%
**How do you score an ELL response that would be incorrect for a monolingual speaker?**		
Marked as correct	149	60.08%
Marked as incorrect	99	39.92%
**Do you feel your response to the prior question is consistent with other professionals at your school?**		
No	25	10.08%
Not sure	86	34.68%
We were not trained on this	57	22.98%
Yes	80	32.26%
**Do you feel you understand the difference between language delay vs. language difference due to ELL status?**		
No	8	3.23%
Not sure	24	9.68%
We were not trained on this	42	16.94%
Yes	174	70.16%
**Does your understanding of ELL impact how you mark certain responses during screening?**		
No	46	18.55%
Not sure	54	21.77%
Yes	148	59.68%
**What benefits have you observed from implementing the literacy screener?**		
Early identification of students at risk	16	12.31%
Improved differentiation in instruction	3	2.31%
Targeted interventions	4	3.08%
Enhanced parent communication	6	4.62%
Early identification, targeted interventions	15	11.54%
Early identification, enhanced parent communication	6	4.62%
Improved differentiation, targeted interventions	5	3.85%
Targeted interventions, enhanced parent communication	2	1.54%
Early identification, improved differentiation, targeted interventions	12	9.23%
Early identification, improved differentiation, enhanced parent communication	5	3.85%
Early identification, targeted interventions, enhanced parent communication	8	6.15%
Improved differentiation, targeted interventions, enhanced parent communication	5	3.85%
Early identification, improved differentiation, targeted interventions, enhanced parent communication	43	33.08%
*No response (out of total sample)*	118	47.58%

### Summary of expert responses

All three literacy specialists/SLPs disagreed with the statement that English Language Learners (ELLs) cannot be reliably screened for reading difficulties. When asked how they score students with dialectical or language differences, all three reported marking the response as correct rather than penalizing for linguistic variation. Each specialist felt confident in distinguishing between a language delay and a language difference due to ELL status, and all confirmed that their understanding of ELLs impacted how they marked certain responses during screening (e.g., “I’ve been doing it for years. I’m unsure if I’m grading kids with accents (Hispanic, bilingual) correctly”).

Regarding data interpretation and screening practices, all three specialists reported feeling extremely confident in interpreting literacy screening data. However, their approaches to administration adjustments varied—one reported never adjusting procedures, another adjusted sometimes, and one adjusted always based on student needs. Similarly, retesting students due to doubts about accuracy ranged from rarely to sometimes across specialists. All three specialists believed that screening results allow for timely decision-making, though one was unsure. In terms of intervention, two specialists estimated that 10–30% of screened students received proper intervention, while one was uncertain. The most frequently cited benefits of implementing the literacy screener included early identification of students at risk, enhanced communication with parents about student progress, and, for some, more targeted literacy interventions. One specialist also noted that screening data allowed for tracking cohort-level foundational skills and identifying instructional gaps over time.

### Summary of educator responses

#### Data interpretation and intervention confidence

Although most responders (76%) felt confident in interpreting data from the screener, only 38% felt confident in implementing tiered intervention based on literacy screener data, and less than half (44%) indicated that a systematic problem-solving process exists for developing intervention plans for struggling readers. Similarly, only half of respondents (48%) believed that all students had equal opportunities to qualify for intervention following literacy screening. Most respondents (70%) indicated that screening results supported timely decision-making, but only 44% reported having a systematic, formal problem-solving process for creating intervention plans after screening. Critically, only 48% believed that all eligible students in their schools had *equal* opportunities to qualify for interventions.

### ELL student assessment challenges

The majority of respondents (92%) reported that ELL students were screened in their schools, yet 22% believed that these students could not be reliably identified for literacy difficulties. Significantly, 40% of respondents incorrectly marked linguistic variations due to ELL status as incorrect, and 29% struggled to distinguish between language differences and language delays. The majority (60%) reported that their understanding of ELL status influenced how they administered and scored the screener.

### Screening protocol flexibility and accuracy concerns

Adjustments to screening procedures varied across respondents with only a minority (12%) strictly following standardized protocols. The need for retesting also differed, with half of respondents (49%) having to reassess the child due to concerns about accuracy. Educators varied in their confidence levels using screening data for instruction: 38% reported feeling “quite confident” or “extremely confident,” 15% were “somewhat confident,” while the remaining 19% indicated they were only slightly confident or less.

### Turnover to intervention and systemic barriers

Screening turnover to intervention rates varied, with a large proportion of educators reporting challenges in ensuring timely support due to inconsistent MTSS implementation (25%), delays in decision-making (28%), and limited instructional resources (3%). Despite these obstacles, the majority recognized benefits of literacy screening (48%), citing early identification of at-risk students (42%), improved instructional differentiation (25%), more targeted interventions (33%), and enhanced communication with parents regarding student progress (27%).

### Need for diagnostic tools and intervention guidelines

Open-ended responses reinforced the need for clearer intervention guidelines and stronger alignment between screening outcomes and available support services. Educators emphasized frustration with current screening approaches, noting “we need less screeners and more diagnostic tools—teachers need to know exactly where the literacy journey broke down and how to create the plan to close the gap,” and “Administering an online screener with kids in grades K-2 felt very developmentally inappropriate. They have not yet learned how to use computers sufficiently enough to gather accurate data” (SI Table 6). Many expressed concerns about the limitations of single assessments, with responses stating “I don’t like judging kids on one test. Not all kids test well. Some get extremely nervous. And some don't give a flip and click, click, click” (SI Table 16).

### ELL assessment training and protocol concerns

Open-ended responses emphasized concerns about insufficient training in assessing ELL students, with educators expressing uncertainty such as (SI Table 9). Educators expressed frustration with rigid screening protocols, noting “test is English only…accent unfamiliar to many students” and “computer-based test, so teachers can’t adjust for ELL students” (SI Table 17), and identified systematic barriers, with one noting “There is no way to correct answers for students with language differences” (SI Table 13).

### Technology infrastructure and software improvements

Technology infrastructure improvements represented another major area of educator recommendations (SI Table 12). Respondents stressed the necessity for enhanced hardware to facilitate smoother screening implementation. Connectivity issues prompted calls for “better wifi connection.” Screen size was also identified as problematic, with one educator noting “some of our student tech have small screens. When all 4 answers are not visible on the screen the kids don’t always know to scroll. Computers with larger screens would be great” (SI Table 12). The preference for tablets was particularly pronounced among educators working with early elementary students, with many noting that they “would really like to have tablets for our K/1 students” because “using chrome books and having to use a mouse or trackpad to drag and scroll is often difficult” (SI Table 12). Software improvements emerged as equally important, with educators frequently recommending adaptive, AI-driven assessments, with one requesting “a true application that learns from student answers and builds from their pattern on what to do next so it can attach to current knowledge and keep building,” automated scoring systems, and user-friendly interfaces to enhance usability and accuracy. Many expressed enthusiasm for technological solutions that could reduce administrative burden, suggesting that it “would be nice if computer scored oral responses from students” and noting technical limitations where “the mic does not pick up all students’ voices” and requesting that “mics be more sensitive to student voices.”

## Discussion

In this study, we conducted a comprehensive survey of randomly selected educators to identify implementation factors that may influence the accuracy of risk identification. Universal screening tools serve a critical function in early literacy instruction by helping identify students who may require additional support to succeed in the general education setting, monitoring progress over time, and guiding data-driven decision-making for intervention planning. This screening process enables a preventative and data-driven approach as a crucial first step toward delivering the supports that will help children develop their reading skills in the critical first years of school. Identifying these on-the-ground barriers and facilitators influencing correct identification of children's literacy needs could inform more effective implementation strategies and, ultimately, enhance student outcomes.

Although there is some variability across federal policies and local guidelines that dictate the choice of a screener and how screening will be conducted at schools, most variability in the policy to intervention pipeline arises at the implementation level. Analysis of state dyslexia legislation has revealed considerable consistency in the main building blocks of universal screening policy, including requirements for evidence-based assessment tools, mandated screening timelines, and intervention frameworks (Gearin et al., 2022). However, the translation of these consistent policies into classroom practice introduces multiple sources of variability that can significantly impact screening accuracy and student outcomes. Variability in the implementation of screening can significantly impact outcomes, leading to both over- and under-identification of students and ultimately undermining the intended purpose of early screening (Cummings et al., 2019; Poulsen, 2018).

In this study, we provide a snapshot of how literacy screeners are implemented across diverse schools in the United States, drawing directly from the perspectives of those responsible for their administration. We found substantial variability across the EPIS dimensions. Specifically, although the choice of screener and the frequency of its administration were consistent with the best practices guidelines, gaps in training, inconsistent administration procedures and environments, confusion around scoring, and unclear pathways to intervention, all are factors threatening to diminish screening effectiveness. In the following sections, we detail these challenges and discuss their potential implications.

### Exploration: readiness and contextual factors

The respondents in our study represented a diverse group of educators across various professional roles, levels of experience, school district types, and geographic regions within the United States, including linguistically and socioeconomically diverse districts. Most respondents administered screeners aligned with best practices and as specified by legislation. Screeners were administered three times a year and were research-backed off-the-shelf standardized tools. Furthermore, the majority of the tools included the recommended constructs of phonological awareness and letter-sound knowledge, both of which are well-established predictors of reading difficulties (Ozernov-Palchik et al., 2017). However, language-based components, which are particularly important for students with comprehension difficulties (Bao et al., 2024) or those from diverse linguistic backgrounds (Gersten et al., 2007), were present in fewer than half of the screeners used. These gaps indicate that while screening practices align with research-based recommendations in key areas for literacy screening, they may not fully capture the multifaceted nature of risk, particularly in cases where deficits extend beyond foundational decoding skills.

A notable challenge apparent in educator reports is the widespread use of multiple screeners within districts. Across responses, 60 unique combinations of screeners were reported. While using multiple screeners can provide a more comprehensive picture of students’ skills, it also places significant demands on educator training and undermines fidelity in implementation. Educators tasked with administering multiple assessments may struggle with inconsistencies in scoring, interpretation, and alignment with intervention practices, particularly if professional development is insufficient or if screeners vary in design and purpose.

### Preparation: capacity building and training

Proper training is central to the reliable administration of universal literacy screeners (Grisham-Brown et al., 2008). State-level policies reinforce this point by requiring in-service training and continuous support for educators (Gearin et al., 2020) and widely used screening tools report their classification accuracy based on data collected under standardized conditions with well-prepared examiners (NCII, n.d.). When educators lack sufficient preparation, however, the fidelity of the screening process may be compromised, increasing the risk of misidentifying students who require additional reading support. Previous work indicates that even modest deviations from standardized administration protocols or minor scoring errors can significantly alter students’ results (Christ et al., 2013; Derr-Minneci & Shapiro, 1992; Reed & Sturges, 2012). Notably, administration and scoring-related factors have been shown to account for between 16% (Cummings et al., 2014) and 57% (Christ et al., 2012, 2013) of the variance in student score changes from pretest to posttest. Even in controlled research settings, maintaining high fidelity remains difficult; one intervention study excluded 8% of its dataset due to uncorrectable administration errors, and 91% of the remaining data still required corrections (Reed & Sturges, 2013).

Extensive training—encompassing initial instruction, opportunities to practice administration, regular feedback, and periodic reliability checks—has been shown to improve both scoring accuracy and procedural adherence (Grisham-Brown et al., 2008; Stitt et al., 2003). Beyond administration fidelity, professional development fosters educators’ capacity to interpret screening outcomes, refine instructional choices, and tailor interventions based on individual student needs. Research on professional development in data-based decision-making yields moderate to large effect sizes for teacher knowledge (*g* = 0.57; Gesel et al., 2021) and positive, albeit smaller, gains in student outcomes (*g* = 0.31; Shanahan et al., 2024).

Despite these established best practices, the present findings reveal high variability in how educators are trained and supported. Most participants indicated receiving fewer than three hours of total instruction, a level that appears insufficient given the training demands of commonly used screeners [NCII, n.d.]. Moreover, only a small proportion had access to ongoing feedback or coaching, and fewer than half were satisfied with the training they received. Half of the respondents did not have the chance to practice administration before testing their first student, likely undermining both confidence and accuracy. Among the educators who did practice, many noted receiving no feedback at all, highlighting a critical gap in support mechanisms. Consequently, fewer than half felt confident in their assessment administration skills, with some expressing confusion over key procedures that influence accurate scoring and interpretation.

These findings collectively demonstrate the critical importance of implementing comprehensive, multi-faceted professional development for universal screening programs. Effective training must extend beyond single-session workshops to encompass sustained support through hands-on practice opportunities, ongoing expert feedback, and systematic calibration procedures—elements that are essential for maintaining the psychometric integrity of screening data (Johnson et al., 2006). This systematic approach to professional development serves a dual purpose: it enhances the technical accuracy of risk identification while simultaneously building educators’ procedural knowledge and implementation fidelity. Ultimately, such rigorous preparation ensures that screening results can reliably inform the design and deployment of targeted literacy interventions, thereby strengthening the evidence-based decision-making process that is fundamental to effective reading instruction.

### Implementation: fidelity, barriers, and adaptations

In addition to administrator-level factors affecting assessment accuracy, various environmental and logistical challenges also play a role in screening fidelity. These implementation factors include noise levels, audibility issues, interruptions, and technical difficulties which were commonly reported by respondents as factors that impacted test administration. These factors can play an important role in maintaining child engagement during the screening process (Elliott 2002) and in how accurately child responses reflect their pre-literacy skills rather than noise-induced errors. For example, there are significant associations between speech-in-noise perception and phonological abilities (Eccles et al., 2021; Ozernov-Palchik et al., 2022), suggesting that at-risk children may be disproportionately affected by background noise.

Educators also identified assessment modality (paper-and-pencil versus digital formats) as a potentially influential factor in screening performance, particularly among early elementary students whose exposure to digital assessment platforms varies considerably. However, this concern may reflect practitioner perceptions rather than empirical evidence, as research examining modality effects in kindergarten through third-grade populations has yielded mixed findings (Hare et al., 2024). A direct comparison of mCLASS: DIBELS 8th Edition found high concordance between tablet-based and paper–pencil formats, though the digital version produced more consistent scoring due to audio recording and automated playback capabilities (Wang et al., 2021). Given increasing educational technology integration, future investigations should systematically examine educator-level and student-level moderators that may influence the comparative effectiveness of digital screening instruments.

Consistent with previous findings, various aspects of the screening process, including preparation, administration, and scoring, placed considerable demands on educators’ time (Fohlin et al., 2021; Komesidou et al., 2022). According to educator reports, time constraints emerged as a significant barrier, affecting both logistical scheduling, educators’ work-life balance, and student engagement during screening sessions. This aligns with considerable evidence documenting how teachers work unpaid overtime and how these increased demands contribute to overall dissatisfaction and burnout among educators (RAND Corporation, 2023).

To address these time-related implementation challenges, research supports the use of School-Wide Assessment Team (SWAT) approaches, where dedicated teams rather than individual classroom teachers assume primary responsibility for screening administration (Barrett et al., 2024). The SWAT model offers a dual benefit: it concentrates assessment expertise in trained team members, thereby improving fidelity, while simultaneously reducing the opportunity cost of lost instructional time when classroom teachers must leave their students to conduct screenings. This systematic approach may be particularly valuable given our findings that many educators felt inadequately prepared for screening administration, suggesting that both training quality and time efficiency could be enhanced through centralized assessment teams.

While scoring was not identified as the most time-consuming aspect of screening in educator responses, the open-ended feedback suggested a continued need for more automated scoring processes to enhance efficiency and reduce administrative burden. Automated scoring systems, particularly those utilizing speech recognition technology, could offer significant advantages for large-scale screening implementation by enabling rapid, objective data collection and analysis while potentially minimizing human error (Bailly et al., 2022; Sabatini et al., 2023). Such systems may streamline the assessment process and could allow educators to redirect their time and expertise toward the more critical tasks of interpreting results and implementing targeted instructional interventions based on screening data.

Finally, we investigated whether sociodemographic factors influenced the barriers reported in universal screening implementation, hypothesizing that schools serving a higher proportion of students eligible for free or reduced-price lunch would encounter greater resource constraints. Consistent with this hypothesis, educators from lower SES schools reported a significantly higher frequency of technical difficulties (occurring in 60% of screening sessions) compared to educators from higher-SES schools (occurring in 40% of screening sessions). This result aligns with prior research demonstrating that low-income communities often face increased technological barriers when implementing digital or online educational interventions (Benda et al., 2020; Campbell & Goldstein, 2021).

Our findings suggest that technical difficulties remain a significant barrier to screening implementation, disproportionately affecting lower-SES schools. Interestingly, educators from lower-SES schools were more likely to report that screening did not take time away from other responsibilities, which may reflect differences in scheduling structures or integration of screening into existing routines. These results highlight the importance of providing targeted technical support to schools with fewer resources, while recognizing that time burden may not be the primary barrier to implementation in these settings.

### Sustainment: long-term viability and impact

Although collecting accurate data represents the critical first step for the students who are at risk for experiencing severe reading difficulties, such data become inconsequential without proper interpretation and systematic decision-making processes that ensure timely intervention. Sustainment-level factors therefore focus on ensuring that all students continue to receive adequate instruction and equitable educational opportunities over time. Two main themes emerged from the data analysis: whether screener results effectively inform data-driven instructional decision-making processes, and equity concerns regarding accurate assessment of diverse learners and equal access to intervention.

Early screening laws were motivated by research evidence that interventions are most effective in the earliest K–2 grades and become less effective over time (Gersten et al., 2020; Neitzel et al., 2022; Slavin et al., 2011; Suggate, 2010; Swanson et al., 1999; Wanzek et al., 2016, 2018). However, successful implementation depends on systematic processes that enable educators to translate assessment results into targeted instructional adjustments through instrumental use of data rather than intuitive approaches (Espinas & Fuchs, 2022; Fuchs et al., 2021). Research consistently demonstrates that educators face ongoing challenges in this translation process. While many teachers possess strong conceptual understanding of reading development, they often struggle to apply assessment results to design tailored instruction (Al Otaiba et al., 2019; Binks-Cantrell et al., 2011; Spear-Swerling & Cheesman, 2012). This gap between theoretical knowledge and practical application was evident in our study, as educators expressed limited readiness to develop individualized interventions based on systematic data analysis, despite recognizing the value of screening.

Our findings reveal significant concerns about systematic, data-informed decision-making processes to inform instructional modifications. Nearly half of educators reported lacking systematic, formal problem-solving processes for developing intervention plans, while fewer than half believed all students have equal opportunity to qualify for intervention. These implementation gaps translate into concerning outcomes: only about one-third of respondents indicated that the majority of qualified screening results lead to proper intervention, while approximately half indicated that most qualifying students do not receive appropriate support. This indicates that the screening to intervention pipeline is leaky for most students.

Regarding equity concerns with ELLs, the data reveal widespread uncertainty and inconsistency in assessment practices. While the vast majority of educators administer universal screeners to ELL students, nearly a quarter believe ELLs cannot be reliably screened for reading difficulties. However, this skepticism contradicts extensive research demonstrating that ELLs can be reliably screened using English literacy assessments. Multiple studies have found comparable psychometric properties and similar predictive accuracy for ELLs and English proficient students with foundational reading screeners (Betts et al., 2008; Roehrig et al., 2008). Large-scale research confirms that decision thresholds yield similar predictive utility for both groups, with differences in optimal cut scores being minimal—typically requiring only a 5-point adjustment lower for ELLs in kindergarten, while using identical thresholds for grades 1–3 (Cummings et al., 2021). Studies examining diagnostic accuracy found no differences in area under the curve values, indicating comparable overall screener performance between ELLs and English proficient students (Hosp et al., 2011).

These findings suggest that the discrepancies in screening that disproportionately affect ELLs arise not from inadequate psychometric properties of the screeners themselves, but from insufficient training in administration and interpretation. Most educators mark ELL responses as correct even when they would be incorrect for monolingual speakers. Additionally, a substantial proportion of educators reported receiving no training on distinguishing between language delays and language differences due to ELL status, while most acknowledged that their understanding of ELL issues impacts their scoring decisions. These training gaps, rather than screener limitations, appear to drive the inconsistent scoring practices that can lead to misidentification. Emerging research documents persistent inequities in special education services based on student race/ethnicity and socioeconomic status (Cassidy et al., 2023; Darrington et al., 2022; Papandrea et al., 2023; Skrtic et al., 2021), suggesting that inadequate preparation for screening diverse learners, beyond inherent assessment bias, may contribute to these ongoing disparities.

### Summary and future directions

Our findings reveal the complex nature of implementing universal screening in real-world settings, which may partly explain why evidence-based policies have not consistently yielded large-scale, sustainable improvements in children’s reading outcomes. In this discussion, we explore strategies to address these implementation challenges, focusing on integrating key implementation factors into state law and developing the necessary infrastructure to support effective implementation.

#### Embedding implementation factors into state legislation

Key legislative considerations are critical for enhancing the fidelity and overall effectiveness of universal literacy screening initiatives. To bridge the gap between policy and practice, state laws must do more than mandate early identification and what assessments to use; they must also incorporate clear, evidence-based implementation requirements. This includes establishing standardized training protocols and ongoing professional development for educators, ensuring that they are equipped to administer screeners consistently and accurately. There should also be specification of appropriate contexts for administration of assessments, such as designated quiet locations and the use of tools like head-mounted microphones to improve audibility. Additionally, equitable allocation of resources is essential to support the uniform implementation of screening tools across diverse districts. Finally, legislation should require annual, disaggregated reporting of screener outcomes detailing eligibility and intervention provision by student population (i.e., ELL, socioeconomic status, racial minority) to monitor progress and identify gaps in service delivery.

#### Implementation infrastructure beyond legislative mandates

While legislative requirements provide an essential foundation for universal screening, they represent only the starting point in a complex implementation process (Sanetti & Luh, 2019). The translation from statutory mandate to effective practice requires comprehensive implementation infrastructure that extends far beyond what legislation alone can accomplish. This infrastructure must include (1) district-level operational procedures that translate broad legislative requirements into specific, actionable protocols tailored to local contexts; (2) systematic selection processes for screening tools that consider not only technical adequacy but also alignment with educator capacity and student population needs; (3) sustained professional development systems that go beyond initial training to include ongoing coaching, feedback loops, and communities of practice; (4) quality assurance mechanisms including fidelity monitoring and data review processes; and (5) integrated data systems that connect screening results to intervention planning and progress monitoring. The gap between policy adoption and meaningful implementation often stems from insufficient attention to these intermediary structures. Critically, without systematic follow-up and performance feedback, even well-designed screening procedures risk becoming what Noell and Gansle (2006) describe as a “hollow shell”—where meetings are held, assessments are planned, and forms are completed, but the actual implementation never occurs or degrades rapidly.

### Limitations

Several limitations should be considered when interpreting these findings. First, this study relies primarily on educator self-reports, which may not accurately reflect actual implementation fidelity or screening accuracy (Kruger & Dunning, 1999). Second, the survey experienced substantial missing data (29.74% of all data points), primarily due to survey fatigue, which may have introduced systematic bias. This pattern of systematic missingness may have differentially affected responses from educators with varying levels of experience or commitment to screening practices. Third, our sampling approach, while geographically diverse, relied on convenience sampling through professional networks and social media platforms, which may have introduced selection bias toward educators who are more engaged with literacy assessment or have stronger opinions about screening practices. Additionally, we could not verify the identity of respondents or confirm they were legitimate members of the target population, despite including demographic and role-specific verification questions. Finally, while this study identifies numerous sources of implementation variability reported by educators, we did not directly measure the impact of these factors on actual screening accuracy or student outcomes. The relationship between reported implementation challenges and objective measures of screening effectiveness remains an important area for future research.

The persistent challenge of achieving literacy proficiency at scale arises not from a lack of evidence about *what* works, but from a chronic neglect of *how*—and for *whom*—these solutions are implemented in practice. School districts are complex ecologies, each presenting a unique constellation of challenges. Without scientifically evaluating and addressing this diversity, policies mandating evidence-based universal screening remain paradoxically non-universal, grounded more in aspiration than in real-world implementation.

## Supplementary Information

Below is the link to the electronic supplementary material.

ESM 1 (DOCX. 112 KB)

## Data Availability

Data, analysis code, and key materials are publicly available on OSF at https://osf.io/wfr4h/and GitHub at https://github.com/sensein/readnet_survey.
