# Supplementary material for: (Not so) universal literacy screening: a survey of educators reveals variability in implementation
Source: Ann Dyslexia. 2025 Oct 29;75(3):489–523. doi: 10.1007/s11881-025-00342-1 (PMC12662888; doi:10.1007/s11881-025-00342-1)
Supplement: Supplementary file 1 — (DOCX. 112 KB) [file 11881_2025_342_MOESM1_ESM.docx]

**SI-1 Missing Data Patterns**

**Extent and Distribution**

Missing data was substantial, affecting 29.74% of all possible data points (3,614 out of 12,152) across 41 of 49 variables. Individual variables showed missing rates ranging from 3.2% to 83.47%, with four variables exceeding 50% missing data. The dataset exhibited 170 unique missing patterns, indicating complex systematic non-response rather than random item-level missingness.

**Survey Position Effects**

A strong positive correlation emerged between question order and missing rates (Spearman ρ = 0.58, *p* < 0.001), providing clear evidence of survey fatigue as the primary mechanism driving systematic missingness. Missing rates varied systematically by question position: early questions (1-10) averaged 26.1% missing, while later questions (31-49) averaged 40.7% missing, demonstrating systematic survey abandonment patterns related to respondent burden rather than content sensitivity.

**Missing Data Dependencies**

Chi-square independence tests across 819 variable pairs revealed that 86% showed statistically significant dependent missingness (*p* < 0.05), providing strong evidence against Missing Completely At Random (MCAR). The most significant dependencies involved systematic co-occurrence of missing responses across related question blocks, consistent with position-based abandonment rather than content-specific non-response patterns.

**Demographic Independence**

Importantly, missing data patterns showed no significant relationship to demographic characteristics. Survey fatigue effects were consistent across all Free/Reduced Lunch categories, with no significant differences in fatigue effects between socioeconomic groups (*p* = 0.290). This indicates that survey position, not demographic factors, drives the systematic missingness patterns. All demographic groups showed similar fatigue effects (25.1 to 40.1 percentage point drops from early to late questions), suggesting that respondent burden affects participants uniformly regardless of school characteristics.

**SI Table 1: Missing Data Analysis Summary**

| **Analysis Component** | **Method** | **Key Metrics** |
| --- | --- | --- |
| Extent & Distribution | Descriptive statistics | • 29.74% total missing (3,614/12,152 data points); • 41 of 49 variables affected; • 3.2%-83.47% range per variable; • 170 unique missing patterns |
| Survey Position Effects | Spearman correlation | • ρ = 0.58, p < 0.001; • Early questions (1-10): 26.1% missing; • Late questions (31-49): 40.7% missing |
| Missing Dependencies | Chi-square tests (819 pairs) | • 86% showed significant dependencies (p < 0.05); • Systematic co-occurrence across question blocks |
| Demographic Analysis | ANOVA by FRL status | • No significant differences (F p = 0.290); • Consistent fatigue across all groups; • 25.1-40.1% point drop from early to late |

**SI Table 2: Missing Data Summary Statistics**

| **Statistic** | **Value** |
| --- | --- |
| Total Survey Items | 49 |
| Total Respondents | 248 |
| Items with No Missing Data | 8 |
| Items with <20% Missing | 7 |
| Items with 20-50% Missing | 30 |
| Items with >50% Missing | 4 |
| Full Survey Completers | 2 |
| High Completers (80%+) | 138 |
| Moderate Completers (50-79%) | 36 |
| Low Completers (<50%) | 72 |
| Average Questions Answered per Respondent | 34.4 |
| Average Missing Rate per Item | 29.74% |
| Survey Fatigue Correlation | 0.582 |

**Multiple Imputation Methods**

We implemented multiple imputation using the MICE (Multivariate Imputation by Chained Equations) package in R to address the systematic missingness identified in our survey data. Given the strong survey fatigue effects (ρ = 0.58 between question position and missing rates), we incorporated auxiliary variables to model completion patterns, including early, middle, and late question completion rates, overall completion tendency, and survey abandonment indicators. Variables with >80% missing data were excluded from imputation to avoid unreliable estimates. The imputation strategy employed predictive mean matching (PMM) for numeric variables and factors with many levels, logistic regression for binary variables, and polytomous regression for ordinal factors with few levels. To enhance imputation accuracy, we included demographic variables with <10% missingness (State, School Type, District Type, Free/Reduced Lunch status) as predictors in the imputation model. We generated 20 imputed datasets with 15 iterations each to ensure convergence and stability, following best practices for handling Missing at Random (MAR) data. This approach allowed us to preserve the relationships between variables while accounting for the uncertainty introduced by missing data, particularly addressing the position-based missingness pattern rather than assuming random dropout.  However, imputation was unsuccessful for multi-category items including "Mode of training" (8 categories, 27.4% missing) and "Screening turnover to intervention" (4 categories, 44% missing) where sparse data conditions prevented reliable imputation, and for derived variables such as "At least 1 interruption" (35.7% missing) and "At least 1 technical issue" (57.0% missing) that required complete source data for calculation.

**SI-2: More Detailed Survey Responses**

**SI Table 3: State Information**

| **State** | **Frequency** | **Percentage** |
| --- | --- | --- |
| Massachusetts | 81 | 32.7% |
| Colorado | 13 | 5.2% |
| Oklahoma | 11 | 4.4% |
| Washington | 8 | 3.2% |
| Illinois | 7 | 2.8% |
| Michigan | 7 | 2.8% |
| Rhode Island | 7 | 2.8% |
| Texas | 7 | 2.8% |
| California | 6 | 2.4% |
| Oregon | 6 | 2.4% |
| Vermont | 6 | 2.4% |
| Maryland | 5 | 2.02% |
| Georgia | 5 | 2.02% |
| New Hampshire | 5 | 2.02% |
| New York | 5 | 2.02% |
| Alabama | 4 | 1.6% |
| Connecticut | 4 | 1.6% |
| Pennsylvania | 4 | 1.6% |
| Tennessee | 4 | 1.6% |
| Virginia | 4 | 1.6% |
| Kansas | 3 | 1.2% |
| Indiana | 3 | 1.2% |
| Missouri | 3 | 1.2% |
| New Jersey | 3 | 1.2% |
| North Carolina | 3 | 1.2% |
| Ohio | 3 | 1.2% |
| Utah | 3 | 1.2% |
| Arkansas | 2 | 0.8% |
| Idaho | 2 | 0.8% |
| Iowa | 2 | 0.8% |
| Minnesota | 2 | 0.8% |
| Delaware | 1 | 0.4% |
| Florida | 1 | 0.4% |
| Kentucky | 1 | 0.4% |
| Louisana | 1 | 0.4% |
| Maine | 1 | 0.4% |
| Montana | 1 | 0.4% |
| South Carolina | 1 | 0.4% |
| West Virginia | 1 | 0.4% |
| Other | 11 | 4.4% |

**SI Table 4: Screener Information**

| **Rank** | **Screener** | **Frequency** | **Percentage** |
| --- | --- | --- | --- |
| 1 | DIBELS 8th Edition from University of Oregon | 73 | 29.3% |
| 2 | mCLASS from Amplify | 47 | 18.9% |
| 3 | i-READY from Curriculum Associates | 46 | 18.5% |
| 4 | STAR (Early Literacy, Reading and CBM) from Renaissance | 46 | 18.5% |
| 5 | MAP Reading Fluency from NWEA | 29 | 11.6% |
| 6 | The Fountas & Pinnell Benchmark Assessment Systems | 23 | 9.2% |
| 7 | Acadience Reading K, Voyager Sopris Learning | 19 | 7.6% |
| 8 | Fastbridge from Illuminate Education | 17 | 6.8% |
| 9 | EarlyBird from EarlyBird Education | 12 | 4.8% |
| 10 | Other (please specify) | 38 | 15.3% |

**SI Table 5: Most Common Screener Combinations (n=85 multiple screener responses):**

| **Rank** | **Combination** | **Frequency** | **Percentage** |
| --- | --- | --- | --- |
| 1 | DIBELS & i-READY | 9 | 3.6% |
| 2 | DIBELS & MAP | 4 | 1.6% |
| 3 | DIBELS & STAR | 4 | 1.6% |
| 4 | mCLASS & STAR | 4 | 1.6% |
| 5 | DIBELS & mCLASS | 3 | 1.2% |

Note: Based on 249 survey responses reporting 60 unique screener combinations. Individual tool percentages represent usage frequency; totals exceed 100% due to multiple screener use. Single screener responses: n=164 (65.9%); multiple screener responses: n=85 (34.1%). "Other" includes 36 additional tools (e.g., Aimsweb, Istation Reading ISIP, Clay's Observation Survey).

**Table SI-3. Overview of OSF‑hosted materials**

All data, code, and materials are publicly available on OSF at <https://osf.io/wfr4h>. The contents are organized into four folders:

| **Folder** | **Description** |
| --- | --- |
| **Code** | Five R scripts used to process, analyze, and summarize the data: |
|  | - data_imputation_062925.R: Performs data cleaning and imputation of missing values. |
|  | - qual_data_by_group.R: Analyzes open-ended responses by group using thematic coding. |
|  | - Results_summary.R: Aggregates and summarizes results from quantitative analyses. |
|  | - SES_analysis_070325.R: Examines implementation differences across SES strata. |
|  | - survey_analysis022025.R: Main script for analysis of survey responses. |
|  | Scripts are annotated for reproducibility and structured for stepwise execution. |
| **Preprint** | PDF of the manuscript uploaded to OSF Preprints, including abstract, main text, and figures. |
| **Survey materials** | Includes the complete survey instrument, consent form, and administration protocol. |
| **Data** | Contains both raw and cleaned datasets used in the analyses: |
|  | - raw_data/: Direct exports from the survey platform. |
|  | - processed_data/: Cleaned and de-identified data files used in all analyses. |
|  | No README is provided; variable names and coding are documented within the code files. |

**SI-4: Qualitative Tables**

**SI Table 6**: Thematic analysis of responses on training for screener administration

# Survey Question Theme Example Responses Percentage (n)

*If there’s any additional information that you’d like to mention about training for screener administration, please elaborate.*

Inadequate or Superficial Initial Training

Need for Ongoing Training and Support

Data Interpretation and Practical Application Issues

Concerns with Screener Appropriateness or Effectiveness

Reliance on Previous Experience or Self-directed Training

“No onboarding for new hires. . . ” “Initial training was a quick

overview. . . all meaningful learning on my own.”

“The district needs to provide us with ongoing training during school hours!” “We also did a refresh training. . . prior to each benchmarking window.”

“Teachers need to know exactly where the literacy journey broke down. . . ”

“Any more indepth usage of data/analysis is on own. . . ”

“Administering an online screener. . . felt very developmentally inappropriate.” “We need less screeners and more diagnostic tools. . . ”

“Training was part of Master’s program. . . ”

“Training was 12+ years ago. . . but as I use Running Records regularly. . . ”

28% (11)

23% (9)

18% (7)

15% (6)

15% (6)

**SI Table 7**: Thematic analysis of responses on the quality of training provided for screener administration

# Survey Question Theme Example Responses Percentage (n)

*How would you rate the quality of the training provided? If you care to, please elaborate on your previous answer.*

Quality and Relevance Issues

Self-Directed and Prior Experience

Depth and Continuity of Training

Timing and Implementation Issues

Vendor or Sales-Focused Training

“Poor for interpretation of results. . . Lack of understanding on test construction.” “were trained how to set up screeners and where to retrieve the data, but not on what exactly the data shows, what each category means, or even what is specifically asked of students on the test”

“I mostly just trained myself.” “Graduate training was

excellent. . . training in current school is minimal.”

“Early Bird’s ongoing PD model is one of its biggest strengths!”

“Partner with Root Literacy Design who provides training and coaching throughout the year. . . ”

“Training was too late, over a year after we started. . . ”

“Initial training fine but never any follow-up. . . ”

“Good for selling their product. . . ” “A rep did an overview. . . wouldn’t describe their algorithm.”

36% (14)

28% (11)

26% (10)

15% (6)

13% (5)

**SI Table 8**: Thematic analysis of responses on opportunities to practice and receive feedback for screener administration.

# Survey Question Theme Example Responses Percentage (n)

*If you were given an opportunity to practice administering the literacy screener, what did that entail? Did you receive feedback from an experienced tester on your performance?*

*Please elaborate.*

Self-directed Practice Without Feedback

Trainer or Specialist-Led Feedback and Practice

Simulation-based Practice

Peer Practice and Mutual Feedback

Structured Practice Sessions (I do, we do, you do)

“No feedback.”

“Solo practice, student perspective.”

“Experienced tester sat with me and gave feedback.”

“Guided practice with literacy specialist modeling.”

“Demo class, no feedback given.” “Used training app on the iPad.”

“met in the library and did a few practice screeners together, graded them, and questions were answered”

“I participated as mock administrator/student with other teachers.”

“I administered while teacher watched, then teacher administered with my feedback.”

“Colleagues with the opportunity to practice administering the screener in a I do, we do, you do format”

23% (13)

21% (12)

18% (10)

16% (9)

9% (5)

**SI Table 9**: Thematic analysis of responses on confidence in administering the literacy screener

| **Survey Question** | **Theme** | **Example Responses** | **Percentage (n)** |
| --- | --- | --- | --- |
| *How confident did* | Confidence from | “I have a masters in reading.” | 28% (14) |
| *you feel to* | Prior Experience or | “I’ve been an interventionist for 30 |  |
| *administer the* | Education | years. . . ” |  |
| *literacy screener?* | Initial Uncertainty | “I was worried I wasn’t doing it correctly | 24% (12) |
| *If you care to,* | and Need for | at first. . . ” |  |
| *please elaborate.* | Practice | “It always takes some practice getting |  |
|  |  | into the routine. . . ” |  |
|  | Role or | “The I-Ready screener is an app and the | 18% (9) |
|  | Context-Specific | teacher is not involved. . . ” |  |
|  | Limitations | “Our literacy coaches give the screener.” |  |
|  | Administrative and | “I’ve been doing it for years. I’m unsure if | 16% (8) |
|  | Scoring Challenges | I’m grading kids with accents (Hispanic, |  |
|  |  | bilingual) correctly” |  |
|  |  | “MCLASS particular ways you must |  |
|  |  | mark mistakes. . . ” |  |
|  | Self-Reliance and | “Had to do my own digging to figure it | 14% (7) |
|  | Independent | out. . . ” |  |
|  | Learning | “The test was pretty easy to figure out on |  |
|  |  | my own. . . ” |  |

**SI Table 10**: Thematic analysis of responses on how the assessment environment influenced student comfort and performance

# Survey Question Theme Example Responses Percentage (n)

*Did the environment in which the screener was administered affect student comfort or performance?*

*Please elaborate.*

Familiar Environment (No issues)

Distractions in Shared or Busy Spaces

Unfamiliar Environment Causing Discomfort

Comfort-enhancing Strategies

Unfamiliar Adults Affecting Student Comfort

“No, they were happy to come and it was actually less distracting.”

“conducting this assessment in person provides accurate results”

“Distractions in the hallway. . . other kids pass by.”

“Resource room shared and can be loud.”

“Yes–new space, new distractions. Apprehension due to novel environment.” “Students interested in surroundings they hadn’t seen before.”

“I use calming colors and stuffies in my office.”

“Orient the child to the environment. . . answer questions before we begin.”

“Yes–unknown adult administering in noisy hallway.”

“Students not familiar with the adult. . . took longer to engage.”

38% (23)

28% (17)

15% (9)

10% (6)

8% (5)

**SI Table 11**: Thematic analysis of responses on how screening duration impacts implementation

# Survey Question Theme Example Responses Percentage (n)

*How does the duration of the screening process impact its implementation, particularly in terms of scheduling and ensuring the child’s engagement throughout the screening?*

Impact on Student Engagement

Scheduling and Instructional Time Pressures

Administrative and Logistical Challenges

Individual Differences and Special Needs

Benefits of Short/Efficient Screening

”Many children need multiple sessions due to lack of attention.”

”get very bored which negatively impacts how well they do”

”Two-week window is stressful. . . scramble to do screenings.”

”It can be challenging to find screening time with a busy caseload.”

”Before subs it could be difficult to complete assessments.”

”Materials need to be prepared ahead for smooth transitions.”

”Students with language disorders, autism, ADHD sometimes take longer.” ”Some kids sustain attention better than others.”

”children get bored taking iReady so they will often just click through”

28% (14)

24% (12)

18% (9)

16% (8)

14% (7)

**SI Table 12**: Thematic analysis of responses on desired technology enhancements for literacy screening practice

| **Survey Question** | **Theme** | **Example Responses** | **Percentage (n)** |
| --- | --- | --- | --- |
| *If you could ask for any technology to enhance your practice, what would it be?*  *Please elaborate.* | Device  Enhancements | ”iPads available to all teachers.”  ”Updated chromebooks.”  ”would really like to have tablets for our K/1 students”  ”some of our student tech have small screens. When all 4 answers are not visible on the screen the kids don’t always know to scroll. Computers with larger screens would be great”  ”using chrome books and having to use a mouse or trackpad to drag and scroll is often difficult” | 31% (22) |
|  | Improved Audio and Microphone Quality | ”High-quality headphones. . . ” ”Microphones or reliable audio pickup. . . ” ”high quality headphones to allow for students to listen and record their reading”  ”mics be more sensitive to student voices” | 15% (11) |
|  | Enhanced Digital Assessment Tools | ”Would be nice if computer scored oral responses. . . ”  ”A true application that learns from student answers. . . ”  ”More detailed analysis of student errors. . . ” | 13% (9) |
|  | Preference for Non-technological Methods | ”Return to paper/pencil.”  ”Less technology is wanted for K-2!” | 10% (7) |
|  | Better Connectivity | ”Better wifi connection. . . ”  ”networks are not able to handle multiple classes giving screeners at the same time” ”connectivity can be an issue” | 8% (6) |

**SI Table 13**: Thematic analysis of responses on difficulties in scoring the universal literacy screening assessment.

# Survey Question Theme Example Responses Percentage (n)

*Do you ever experience difficulties in scoring the universal literacy screening assessment? If you care to, please elaborate.*

Ease of Automated Scoring

Real-time Scoring Challenges

Task-specific Scoring Difficulties

Student Speech and Response Clarity Issues

Human Variability and Judgment Issues

”The assessment is automatically scored through the system.”

”If a student reads quickly. . . it can be hard to keep up.”

”it is extremely difficult to mark errors in real time on the non-word fluency portion”

”Phoneme Segmentation task and the Nonsense Word Fluency task are difficult to score.”

”there is no way to correct answers for students with language differences” ”Students with articulation problems. . . deviate from task.”

”Some teachers are more lenient than others.”

”Judgment call regarding cut scores.”

38% (10)

19% (5)

15% (4)

12% (3)

8% (2)

**SI Table 14**: Thematic analysis of responses on the impact of family involvement during remote or hybrid screening administration.

# Survey Question Theme Example Responses Percentage (n)

*How does family involvement, particularly for a remote or hybrid mode of administration, during the screening impact the implementation process?*

Limited or No Experience with Remote/Hybrid

Parental Interference or Prompting

Distractions and Environmental Challenges

Inflated or Inaccurate Scores

Parent Understanding and Engagement Issues

“Family not present during screening.” “No hybrid or virtual option.”

“Parents help students by giving answers.”

“Sometimes parents interfere by giving prompts or cues.”

“Constant distraction—TV, siblings, parents interrupting.”

“Parents didn’t understand. . . constant distraction.”

“Scores were extremely inflated.” “parents intervening and making results invalid”

“Parents didn’t understand the importance of the lesson and testing.”

36% (5)

29% (4)

14% (2)

14% (2)

7% (1)

**SI Table 15**: Thematic analysis of responses on whether screener administration and scoring take valuable time away from other responsibilities

# Survey Question Theme Example Responses Percentage (n)

*Does administration and scoring of the screener take*

*valuable time away from your other responsibilities, and if so, which aspects of the assessment are most*

*time-consuming?*

Perceived Value and Productivity

Administrative and Logistical Responsibilities

Assigned to Others or Automated

Improved Efficiency Compared to Past

Acceptable Interruption of Other Activities

Impact on Personal Time

“Somewhat—but the time is worth it. The info is valuable.”

“The time used on assessments is valuable to instruction.”

“Administering and data analysis are most time consuming.”

“I am responsible. . . to prepare materials, make schedules, assess, score, interpret results.”

“Someone else administers and scores.” “It’s all computerized.”

“Our current screener takes less than half the time our previous screener took.” “DIBELS used to take time, now through mClass there’s no scoring time.”

“We stop reading interventions. . . it takes some time.”

“Daily activities still have to stay on schedule.”

“I do a lot of this at home so it takes away from my family.”

35% (6)

18% (3)

18% (3)

12% (2)

12% (2)

6% (1)

**SI Table 16: Thematic analysis of responses on challenges encountered during administration of the literacy screener**

| **Survey Question** | **Theme** | **Example Responses** | **Percentage (n)** |
| --- | --- | --- | --- |
|  | Teacher Resistance | “Resistance and lack of understanding | 21% (12) |
| *What challenges* | and | from teachers.” |  |
| *(if any) have you* | Misunderstanding | “Many teachers do not understand how to |  |
| *encountered while* |  | use data.” |  |
| *administering the* | Logistical and | “Getting sub coverage to complete | 19% (11) |
| *literacy screener?* | Resource | assessments.” |  |
|  | Constraints | “No good space to test. School very |  |
|  |  | crowded.” |  |
|  | Concerns About | “5 and 6-year-olds too young to be tested | 18% (10) |
|  | Validity and | on a computer.” |  |
|  | Appropriateness | “Results unclear, do not provide useful |  |
|  |  | info...” |  |
|  | Administrative and | “Resistance from administrators.” | 16% (9) |
|  | Systemic Issues | “Lack of response from admin.” |  |
|  | Communication and | “Parent reports could be more | 10% (6) |
|  | Interpretation | parent-friendly.” |  |
|  | Challenges | “Anticipate parent misunderstanding.” |  |
|  | Scoring and | “Extremely difficult to mark errors in real | 9% (5) |
|  | Procedural | time on NWF.” |  |
|  | Challenges | “Words not following taught rules.” |  |
|  | Student Engagement | “I don’t like judging kids on one test. Not | 7% (4) |
|  | and Behavior Issues | all kids test well. Some get extremely |  |
|  |  | nervous. And some don’t give a flip and |  |
|  |  | click, click, click” |  |

**SI Table 17: Thematic analysis of responses on the mode of administration**

| **Survey Question** | **Theme** | **Example Responses** | **Percentage (n)** |
| --- | --- | --- | --- |
| *How does the* | Advantages of | ”Being in the room is important in order | 27% (13) |
| *mode of* | In-person | to observe the student during the |  |
| *administration* | Administration | administration of the screener” |  |
| *impact the* |  | ”Conducting this assessment in person |  |
| *screening process?* |  | provides accurate results.” |  |
| *If you care to,* | Technical and | ”Networks not able to handle multiple | 19% (9) |
| *please elaborate.* | Logistical Issues | classes...” |  |
|  |  | ”Connectivity can be an issue.” |  |
|  | Time-Intensity and | ”Had to administer each test | 15% (7) |
|  | Staffing Challenges | individually...very time-consuming.” |  |
|  | Validity and | ”It would be next to impossible to | 15% (7) |
|  | Accuracy Concerns | administer the face-to-face portion of the |  |
|  |  | screener remotely and it still be accurate.” |  |
|  |  | ”During pandemic, we did remote |  |
|  |  | testing... students were more attentive |  |
|  |  | and less distracted on campus” |  |
|  | Environmental | ”Distractions and insufficient time...” | 13% (6) |
|  | Factors and | ”Things always happen while giving the |  |
|  | Distractions | screener because it’s in the classroom.” |  |
|  | Language and | ”Test is English only. . . accent unfamiliar | 13% (6) |
|  | Accessibility Issues | to many students.” |  |
|  |  | ”Computer-based test, so teachers can’t |  |
|  |  | adjust for ELL students.” |  |
